# Predictive Modeling of 30-Day Emergency Hospital Transport of Patients Using a Personal Emergency Response System: Prognostic Retrospective Study

**DOI:** 10.2196/medinform.9907

**Published:** 2018-11-27

**Authors:** Jorn op den Buijs, Mariana Simons, Sara Golas, Nils Fischer, Jennifer Felsted, Linda Schertzer, Stephen Agboola, Joseph Kvedar, Kamal Jethwani

**Affiliations:** 1 Philips Research Eindhoven Netherlands; 2 Partners HealthCare Pivot Labs Partners HealthCare Boston, MA United States; 3 Department of Dermatology Harvard Medical School Boston, MA United States; 4 Philips Lifeline Framingham, MA United States; 5 Department of Dermatology Massachusetts General Hospital Boston, MA United States; 6 Partners Connected Health Partners HealthCare Boston, MA United States

**Keywords:** accountable care organizations, decision support techniques, emergency medical dispatch, machine learning, population health

## Abstract

**Background:**

Telehealth programs have been successful in reducing 30-day readmissions and emergency department visits. However, such programs often focus on the costliest patients with multiple morbidities and last for only 30 to 60 days postdischarge. Inexpensive monitoring of elderly patients via a personal emergency response system (PERS) to identify those at high risk for emergency hospital transport could be used to target interventions and prevent avoidable use of costly readmissions and emergency department visits after 30 to 60 days of telehealth use.

**Objective:**

The objectives of this study were to (1) develop and validate a predictive model of 30-day emergency hospital transport based on PERS data; and (2) compare the model’s predictions with clinical outcomes derived from the electronic health record (EHR).

**Methods:**

We used deidentified medical alert pattern data from 290,434 subscribers to a PERS service to build a gradient tree boosting-based predictive model of 30-day hospital transport, which included predictors derived from subscriber demographics, self-reported medical conditions, caregiver network information, and up to 2 years of retrospective PERS medical alert data. We evaluated the model’s performance on an independent validation cohort (n=289,426). We linked EHR and PERS records for 1815 patients from a home health care program to compare PERS–based risk scores with rates of emergency encounters as recorded in the EHR.

**Results:**

In the validation cohort, 2.22% (6411/289,426) of patients had 1 or more emergency transports in 30 days. The performance of the predictive model of emergency hospital transport, as evaluated by the area under the receiver operating characteristic curve, was 0.779 (95% CI 0.774-0.785). Among the top 1% of predicted high-risk patients, 25.5% had 1 or more emergency hospital transports in the next 30 days. Comparison with clinical outcomes from the EHR showed 3.9 times more emergency encounters among predicted high-risk patients than low-risk patients in the year following the prediction date.

**Conclusions:**

Patient data collected remotely via PERS can be used to reliably predict 30-day emergency hospital transport. Clinical observations from the EHR showed that predicted high-risk patients had nearly four times higher rates of emergency encounters than did low-risk patients. Health care providers could benefit from our validated predictive model by targeting timely preventive interventions to high-risk patients. This could lead to overall improved patient experience, higher quality of care, and more efficient resource utilization.

## Introduction

### Background

With the worldwide increase in the elderly population [1], chronic diseases and associated health care utilization, such as costly emergency department (ED) visits and subsequent hospitalizations, are also on the rise. In the United States, nearly 40% of patients making ED visits arrived by emergency ambulance transport [2,3], and 80% of unscheduled hospital admissions were through the ED [4]. Preventing avoidable ED visits and admissions among elderly patients is becoming a global priority [5,6], since emergency hospitalizations may be particularly distressing for older people and have been associated with adverse events such as hospital-acquired infections, loss of functional independence, and falls [7].

Various telehealth programs have been demonstrated to be effective in reducing readmissions, ED visits, and mortality for patients with congestive heart failure (CHF), stroke, and chronic obstructive pulmonary disease (COPD) [8]. For instance, a large nonprofit health care system in the United States introduced a home-based telehealth program that lasts 30 to 60 days after discharge from hospital, targeting the top 5% high-cost patients with multiple chronic conditions [9]. In addition, our recent work unveiled cost-saving opportunities by managing the patients in the lower-cost segments that are at risk of becoming more costly in the long term, such as beyond institutional settings and 30- to 60-day telehealth services [10]. Population health management programs may benefit from data analytics that integrate home monitoring devices to monitor patients after discharge and find out whether they have issues before ED utilization. These devices include personal emergency response systems (PERSs), which can help older adults get immediate assistance when a serious home-based accident occurs and where delayed response may result in preventable ED utilization [11]. Furthermore, our preliminary findings suggested that predictive analysis using PERS data could be useful for identifying patients at high risk for imminent emergency health care utilization [12].

**Figure 1:**
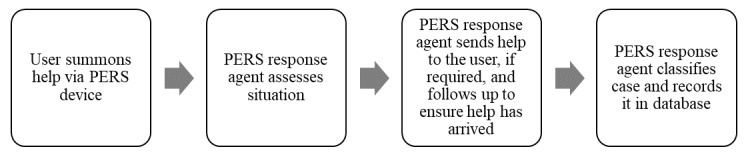
Overview of the personal emergency response system (PERS) process and data collection.

A PERS service enables older adults to get help in a situation that potentially requires emergency transport by ambulance to the hospital, such as a sudden worsening of their chronic condition or a fall [13,14]. PERS is a widely used wearable technology with a help button that is worn as either a bracelet or a pendant. Patients may press the help button at any time to activate an in-home communication system that connects to a 24/7 call center. The call center associate may contact an informal responder (eg, a neighbor or a family member) or emergency medical services (EMSs) based on the patient’s specific situation and follows up with the patient to confirm that help has arrived. The call center associate records notes from the conversations with the subscribers in an electronic record and classifies the type, situation, and outcome of the case (Figure 1). In combination with user enrollment data, such as demographic, caregiver network, and medical condition data, these case data provide valuable information about the patient’s status.

Progress in big-data analysis techniques, such as predictive modeling to identify patients at risk of worsening health conditions, may contribute to cost-effectively reducing potentially avoidable health care utilization [15]. Previous efforts in the field of risk prediction include predictive modeling of hospital readmission [16], repeat ED visits [17,18], and the use of specialized discharge services [19]. For example, the LACE index (length of stay; acuity of the admission; comorbidity of the patient, measured with the Charlson comorbidity index score; and emergency department use, measured as the number of visits in the 6 months before admission) was designed for the prediction of death or unplanned readmissions after hospitalization [20], achieving a predictive performance of the area under the receiver operating characteristic curve (AUC) of 0.68. Another model, HOSPITAL (hemoglobin, discharge from an oncology service, sodium level, procedure performed, index type of admission [urgent vs elective], number of admissions in the last year, and length of stay), is a risk score for predicting 30-day potentially avoidable readmission with a performance of AUC=0.72 as evaluated in 9 hospitals in 4 countries [21]. Yet another study used 1-year retrospective electronic health record (EHR) data to predict 30-day ED revisits, with predictive power of AUC=0.70, in a prospective validation cohort [18]. These predictive models rely only on clinical data collected before or during the clinical encounters or at the time of discharge, but not on data from home monitoring postdischarge. In contrast, PERS services collect information while the patient is back at home. This information includes the details—time stamp, type, situation, and outcome—of incidents such as falls, respiratory issues, chest pain, or general pain, as well as other check-in or social calls [13]. Such events may indicate a decline in patient status, and some patients may eventually request emergency transport to the hospital via the PERS service. Thus, patient decline may be captured earlier with PERS–based prediction models than with models based on clinical data. Therefore, the hypothesis of this study is that the medical alert pattern data collected via the PERS service may be used to predict imminent risk for emergency transport to the hospital.

### Objectives

The objectives of this study were to (1) develop and validate a predictive model of 30-day emergency hospital transport based on PERS data; and (2) compare the model’s predictions with clinical outcomes derived from the EHR, namely outpatient and inpatient emergency encounters. We used a large, deidentified PERS dataset of more than 580,000 individuals to develop and validate the predictive model. For a subpopulation of 1815 patients, we linked PERS and EHR data so that we could compare PERS–predicted risk of hospital transport with observed clinical outcomes.

## Methods

### General Overview

**Figure 2:**
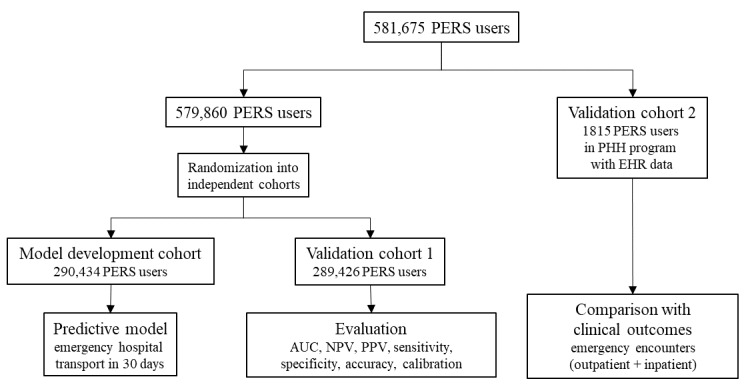
Overview of the study design to develop and evaluate the predictive model of emergency hospital transport. AUC: area under the receiver operating characteristic curve; EHR: electronic health record; NPV: negative predictive value; PERS: personal emergency response system; PHH: Partners HealthCare at Home; PPV: positive predictive value.

In this study, we followed the guidelines by Luo et al for developing and reporting machine learning predictive models in biomedical research [22]. This was a prognostic retrospective predictive modeling study of emergency hospital transport in the next 30 days. Figure 2 presents an overview of the methodology for predictive model development and evaluation. We used retrospective, deidentified data of 581,675 subscribers of a commercial PERS service (Philips Lifeline, Framingham, MA, USA). We developed and validated the predictive model on cohort 1 using data from 579,860 subscribers. For 1815 subscribers (validation cohort 2), who were patients of the Partners HealthCare at Home (PHH; Partners HealthCare at Home, Inc, Waltham, MA, USA) program in the greater Boston, Massachusetts area, we also collected clinical outcomes in the year following prediction to evaluate how prediction of emergency hospital transport compared with rates of outpatient and inpatient emergency encounters. Patients in the model development cohort and both validation cohorts were mutually exclusive.

This study was approved by the Internal Committee on Biomedical Experiments of Philips Research (ICBE-2-16049). Furthermore, we obtained approval for linkage of PERS and EHR records from the Partners HealthCare institutional review board.

### Study Cohorts

#### Model Development Cohort and Validation Cohort 1

We included patients if they were subscribers of the Lifeline PERS service on January 1, 2014 (model development cohort) or February 1, 2014 (model validation cohort 1) and were between the ages of 18 and 100 years. We excluded patients if they appeared to be more than 10 years on the service due to the use of a different back-end system prior to 2004 where some patients may have left the service in the meantime without an indication recorded in the database. Also, patients who left the PERS service for any reason in 30 days from the prediction date were excluded from the analysis. Reasons for leaving the service included death, moving to long-term care, and financial reasons. We excluded patients from the model development cohort and validation cohort 1 if they were part of the PPH program, as we included these patients into validation cohort 2. Furthermore, 3.27% (19,886/606,848) of total patients had missing demographic data and were excluded from the analysis.

The primary PERS data source for model development was the Philips Lifeline database, which contained historical data such as demographics, patients’ living situation and caregiver network, self-reported medical conditions, and medical alert data. We collected data on self-reported medical conditions, including medication allergies, using a custom coding system, which consisted of a drop-down list of common disease categories, such as COPD, CHF, and diabetes. Caregiver network information included the number of responders, the number of people who lived with the patients, and persons to be notified after an incident occurred. Medical alert data from the PERS device included all information gathered during the interactions of the patients with the Lifeline call center associates. Call center associates categorized the calls as incidents or nonincidents. For all calls, the situation (eg, fall, respiratory problems, chest pain, and social call) and the outcome (eg, subscriber okay, responder assistance, and emergency hospital transport) was collected by the associate via custom-made software.

#### Validation Cohort 2

Validation cohort 2 included PERS users who were on the service on February 1, 2014 and received care at PHH, a homecare management service that offers general care as well as specialized services to help patients manage chronic conditions at home. PHH uses a variety of technological innovations to remotely monitor their patients, including the Philips Lifeline PERS, which is a service routinely recommended for elderly or chronically ill patients who are at risk of falls or other health-related emergencies. We excluded patients if they left the PERS service in the 30 days after the prediction date.

The primary data source for comparison of PERS–based predicted risk for emergency hospital transport with clinical outcomes in validation cohort 2 was the Enterprise Data Warehouse, an electronic medical record data repository of hospitals within the Partners HealthCare System. The data include demographic information, medical conditions, and hospital utilization. We combined longitudinal clinical data from the EHR (from February 1, 2014 to January 31, 2015) for 1815 individuals who met the inclusion criteria with the PERS–based predictive score of emergency hospital transport. All data were deidentified before analysis. It should be noted that, in this study, we did not use EHR data to train the predictive model; rather, we compared PERS–based predictions of 30-day emergency hospital transport with clinical observations from the EHR.

### Data Processing

We processed PERS data for input into the predictive model using the statistical programming language R [23]. Different database tables were extracted from an operational PERS database, deidentified, and made accessible to the research team.

One table included subscriber demographics, including age, sex, and region in the United States, subscription type, and enrollment information. A few test accounts, used for demonstration purposes and training of call center agents, were removed prior to the predictive analysis. We used enrollment information to determine which subscribers were active on the prediction date. Subscribers with missing enrollment dates (79,764/1,221,073, 6.53% of all subscribers in the initial data extract) were already deactivated or were pending a PERS installation and were therefore excluded from further analysis. We derived time on the PERS service as the number of days between enrollment and prediction dates.

One table consisted of the subscribers’ caregiver network information. This included the number of responders (eg, son, daughter, or neighbor) and number of emergency service providers (eg, ambulance or fire department) listed by the subscriber at the time of enrollment.

One table included self-reported medical conditions and medication allergies. Medical conditions and medication allergies were recorded at enrollment by the call center agent via a custom-made drop-down list for the purpose of informing EMSs in case of an incident. We extracted the 50 most frequently occurring medical conditions and medication allergies from the database for use in the prediction model. Up to 3 of the most common medical conditions and medication allergies were listed for various categories (Table 1).

**Table 1:** The most common self-reported medical conditions and medication allergies by personal emergency response system subscribers per category (N=581,675).

Category and condition description		Subscribers, n (%)
**Auditory**		
	Hearing loss	66,102 (11.36)
	Hearing aid	20,802 (3.58)
**Cancer**		
	Cancer	11,507 (1.98)
**Cardiovascular**		
	High blood pressure	221,416 (38.07)
	Heart condition	80,113 (13.77)
	History of stroke	41,925 (7.21)
**Endocrine**		
	Diabetes	133,694 (22.98)
	Thyroid disease	10,435 (1.79)
**Medication allergy**		
	Penicillin	85,982 (14.78)
	Sulfa drugs	59,327 (10.20)
	Codeine	42,581 (7.32)
**Musculoskeletal, falls, or movement problems**		
	Cane, crutches, or walker	136,215 (23.42)
	Arthritis	83,565 (14.37)
	History of falls	83,395 (14.34)
**Neurological**		
	Balance problems or unsteady gait	38,672 (6.65)
	Dementia	20,360 (3.50)
	Dizziness or vertigo	17,557 (3.02)
**Psychiatric**		
	Depression	19,788 (3.40)
	Anxiety	12,848 (2.21)
**Pulmonary**		
	Chronic obstructive pulmonary disease	40,868 (7.03)
	Oxygen dependent	28,190 (4.85)
	Asthma	21,905 (3.77)
**Visual**		
	Impaired vision	20,055 (3.45)
	Glasses	15,078 (2.59)
	Macular degeneration	13,207 (2.27)

One table consisted of up to 2 years of historical PERS service use data, which totaled to more than 25 million data points. We categorized cases by case type, situation, and outcome (Table 2). For each case, we constructed 2 features for input to the predictive model, namely frequency and recency. Frequency was the sum of cases experienced by the subscriber prior to the prediction date. Recency was the number of days between the most recent case prior to the prediction date and the prediction date.

**Table 2:** Case types, situations, and outcomes for which frequency and recency features were derived for input into the predictive model. Examples are given per category.

Case category and classification example		Description
**Types**		
	Incident	Case where help is sent to the subscriber
	Accidental	Subscriber accidentally pushed the help button
	Check-in	Social call by subscriber
**Situations**		
	Breathing problems, chest pain, dizziness, fall, illness, other pain	Incident with subscriber experiencing breathing problems, chest pain, dizziness, a fall, illness, or other pain
**Outcomes**		
	Check-in	Social call
	EMS^a^—transport	EMS sent to subscriber’s house—subscriber transported to hospital
	Responder—no transport	Responder sent to subscriber’s house—subscriber not transported to hospital

^a^EMS: emergency medical service.

We derived the dependent variable—having 1 or more emergency hospital transports in the 30 days from the prediction date—from the PERS service use data. As the prediction problem was treated as a classification problem, the dependent variable was rendered binary, with 1 if the patient had 1 or more emergency hospital transports in the 30 days following the prediction date and 0 otherwise.

After preprocessing, we merged the data into 1 large table with 129 columns representing predictive model features and 1 column for the dependent variable. Each of the rows corresponded to 1 PERS user. For 1815 PERS users in the PHH program, clinical outcomes from the EHR were available, and we assigned these to validation cohort 2. We randomly assigned the remaining PERS users on a 1:1 basis to the model development cohort and the validation cohort 1.

### Predictive Model of Emergency Hospital Transport

We then developed a predictive model using a boosted regression trees approach called extreme gradient boosting within R [24]. We chose extreme gradient boosting because it was adopted by more than half of the winning competitions on the Kaggle platform for data science competitions in 2015 [24]. This methodology was also carried out within the commercial PERS–based prediction system CareSage (Philips Lifeline). During initial model development, we also considered logistic regression as a candidate modeling technique but it resulted in significantly lower AUC values in the validation cohort.

The frequency and recency features based on medical alert pattern data are highly skewed. While this may pose a problem for certain predictive modeling methods, the tree-based method we used did not make any assumptions on normality of the features. Monotonic transformations of the features to render them more normal did not affect the predictive performance of the models. We determined variable importance according to the gain, a measure of the relative contribution of the corresponding variable to the predictive model, calculated by taking the improvement in accuracy brought by a variable to the branches it is on. We reduced the number of features by selecting only those with non–zero gain values for the final model, resulting in a total of 121 features in the final model. The boosted regression model also involved tuning hyperparameters of the learning algorithm, such as the number of trees, the maximum depth of the trees, and the learning rate. We achieved this optimization using 5-fold cross-validation on the model development set with the optimization metric determined by the AUC in the test fold.

### Predictive Model Evaluation

We evaluated the discriminatory accuracy of the predictive models using the AUC, which indicates the probability of the predictive model ranking a randomly selected patient with 30-day emergency transport higher than a randomly selected patient without the event. Furthermore, the negative predictive value (NPV) is the percentage of patients not having emergency transport in the group classified as negative, while the positive predictive value (PPV) indicates the percentage of patients having emergency transport in the group classified as positive. We varied the threshold for classifying patients as positive using risk scores above the 90th, 95th, and 99th percentiles such that 10%, 5%, and 1%, respectively, of patients were classified as high risk. For these thresholds, we computed the NPV, PPV, sensitivity, specificity, and accuracy. We derived 95% confidence intervals for performance metrics using a stratified bootstrapping method with 1000 bootstrap replicates.

While we used AUC to assess the ability of the predictive model to discriminate between patients with and without transport, regularization, as done in extreme gradient boosting, may have created bias in the predictive model [22]. Therefore, we also checked the model for calibration—the agreement between predictions made by the model and the outcome—by plotting the observed outcome in ranges of the predicted probabilities (0%-20%, 20%-40%, …, 80%-100%). We also tested goodness-of-fit using the Hosmer-Lemeshow test. This is a systematic way of assessing whether the observed outcomes match the predicted probabilities in subgroups of the model population.

### Statistical Analysis

We tested differences in age between the model development and 2 validation cohorts using analysis of variance followed by Tukey post hoc test. Pairwise comparison between pairs of proportions with Holm correction for multiple testing was applied to the patients’ sex, self-reported medical conditions, and 30-day emergency transport.

To determine how PERS predicted risk for 30-day emergency hospital transport compared with clinical outcomes, we derived the rates of emergency outpatient and inpatient encounters from the EHR at 30, 60, 90, 180, and 365 days following prediction for validation cohort 2. We split the cohort into low, medium, and high risk according to the risk thresholds that corresponded to the 0 to 50th percentile, 51st to 95th percentile, and greater than 95th percentile. The numbers of emergency encounters in these 3 risk groups were normalized as the number of emergency encounters per 100 patients. Emergency encounters were statistically compared using a pairwise Wilcoxon rank sum test.

## Results

### Patient Characteristics and Prevalence of the Dependent Variable

Table 3 presents the characteristics of the model development and both validation cohorts. PERS users were on average 81 years old and most were female (234,817/290,434, 80.85%) in the model development cohort and validation cohort 1. There was no statistically significant difference in age between the model development and validation cohort 1. Average age was slightly, but statistically significantly, younger in validation cohort 2 than in the model development cohort.

The distribution of the number of self-reported conditions was different in validation cohort 2, with only 6.89% (125/1815) of users not reporting any conditions, compared with 20.58% (59,769/290,434) in the model development cohort. One reason may be that these were patients receiving care for 1 or more specific conditions through a home health care program. Regarding the dependent variable of having 1 or more emergency hospital transports in 30 days, 2.20% (40/1815) of patients had emergency hospital transport in the 30 days following the prediction date in validation cohorts 1 and 2. This was not significantly different from the model development cohort (6686/290,434, 2.30%). The ratio of positive to negative classes was 0.024 for the model development cohort and 0.023 for both validation cohorts. According to the EHR data, 509/1815 patients of validation cohort 2 (28.04%) had 1 or more emergency encounters in the year following the prediction date.

### Model Performance

Table 4 details the performance of the predictive model in both validation cohorts for various risk thresholds. The AUC for emergency hospital transport in 30 days was 0.779 (95% CI 0.774-0.785) in validation cohort 1 and 0.766 (95% CI 0.686-0.845) in validation cohort 2. Nonsignificant Hosmer-Lemeshow test results (*P*=.99 for validation cohort 1 and *P*=.78 for validation cohort 2) showed that predicted probabilities matched observed outcomes.

**Table 3:** Patient characteristics and prevalence of the dependent variable in the model development and validation cohorts.

Variable		Model development cohort, (n=290,434)		Validation cohort 1 (n=289,426)					Validation cohort 2 (n=1815)		
				Statistic		*P* value		Statistic		*P* value	
Prediction date		January 1, 2014		February 1, 2014		N/A^a^		February 1, 2014		N/A	
Age (years), mean (SD)		81.3 (11.5)		81.2 (11.5)		.20		79.8 (11.5)		<.001	
Female patients, n (%)		234,817 (80.85)		233,692 (80.74)		.66		1461 (80.50)		.91	
**PERS^b^ self-reported medical conditions, n (%)**											
	0		59,769 (20.58)		61,685 (21.31)		<.001		125 (6.89)		<.001
	1-2		72,067 (24.81)		71,094 (24.56)		.02		656 (36.14)		<.001
	3-4		77,739 (26.77)		76,384 (26.39)		.27		519 (28.60)		.31
	≥5		80,859 (27.84)		80,263 (27.73)		.25		515 (28.37)		.84
Patients with 30-day emergency hospital transport, n (%)		6686 (2.30)		6411 (2.22)		.08		40 (2.20)		.99	
**Patients with ≥** **1 emergency encounters at 3 time points after the prediction date, n (%)**											
	30 days		N/A		N/A		N/A		64 (3.53)		N/A
	180 days		N/A		N/A		N/A		321 (17.69)		N/A
	365 days		N/A		N/A		N/A		509 (28.04)		N/A

^a^N/A: not available.

^b^PERS: personal emergency response system.

**Table 4:** Performance of the predictive model evaluated by the area under the receiver operating characteristic curve (AUC), negative predictive value (NPV), positive predictive value (PPV), sensitivity, specificity, and accuracy using the 90th, 95th, and 99th percentiles as thresholds in the 2 validation cohorts.

Characteristic		Prediction score threshold		
		>90th percentile	>95th percentile	>99th percentile
**Validation cohort 1 (n=289,426)^a^**				
	AUC (95% CI)	0.779 (0.774-0.785)	0.779 (0.774-0.785)	0.779 (0.774-0.785)
	NPV (95% CI)	98.6% (98.6%-98.7%)	98.4% (98.4%-98.4%)	98.0% (98.0%-98.0%)
	PPV (95% CI)	9.6% (9.3%-9.9%)	13.5% (13.1%-14.0%)	25.5% (24.1%-27.2%)
	Sensitivity (95% CI)	43.8% (42.5%-45.0%)	30.5% (29.3%-31.7%)	11.5% (10.7%-12.3%)
	Specificity (95% CI)	90.7% (90.6%-90.8%)	95.6% (95.5%-95.7%)	99.2% (99.2%-99.3%)
	Accuracy (95% CI)	89.6% (89.5%-89.7%)	94.1% (94.1%-94.2%)	97.3% (97.3%-97.3%)
**Validation cohort 2 (n=1815)^b^**				
	AUC (95% CI)	0.766 (0.686-0.845)	0.766 (0.686-0.845)	0.766 (0.686-0.845)
	NPV (95% CI)	98.8% (98.4%-99.1%)	98.3% (98.0%-98.7%)	98.0% (97.8%-98.3%)
	PPV (95% CI)	8.3% (5.9%-10.7%)	9.2% (5.1%-13.9%)	16.7% (4.3%-29.6%)
	Sensitivity (95% CI)	52.5% (37.5%-67.5%)	30.0% (15.0%-45.0%)	12.5% (2.5%-22.5%)
	Specificity (95% CI)	86.9% (85.3%-88.4%)	93.4% (92.2%-94.5%)	98.6% (98.0%-99.1%)
	Accuracy (95% CI)	86.2% (84.4%-87.7%)	92.0% (90.7%-93.2%)	96.7% (96.1%-97.3%)

^a^Hosmer-Lemeshow test: χ^2^_98_=13.1; *P*=.99.

^b^Hosmer-Lemeshow test: χ^2^_8_=4.8; *P*=.78.

PPVs were on the low side due to the low prevalence of 30-day emergency hospital transport, which was around 2.2% in both validation cohorts (Table 3). By increasing the prediction score threshold, PPV increased but at the expense of decreased sensitivity. In validation cohort 1, at a risk score threshold corresponding to the 90th percentile, the predictive model identified 43.8% (95% CI 42.5%-45.0%) of the patients who had emergency transport in the 30 days following the prediction date (sensitivity); however, only 9.6% (95% CI 9.3%-9.9%) of flagged patients had emergency transport in the following 30 days (PPV) at this threshold. At thresholds corresponding to the 95th and 99th percentiles, the sensitivity dropped to 30.5% (95% CI 29.3%-31.7%) and 11.5% (95% CI 10.7%-12.3%), respectively, while the PPV increased to 13.5% (95% CI 13.1%-14.0%) and 25.5% (95% CI 24.1%-27.2%), respectively. This analysis using different thresholds illustrates the trade-off between sensitivity and PPV, that is, trying to find as many positive cases as possible with an acceptable false-positive rate. A similar trade-off between sensitivity and PPV was also observed in validation cohort 1.

**Figure 3:**
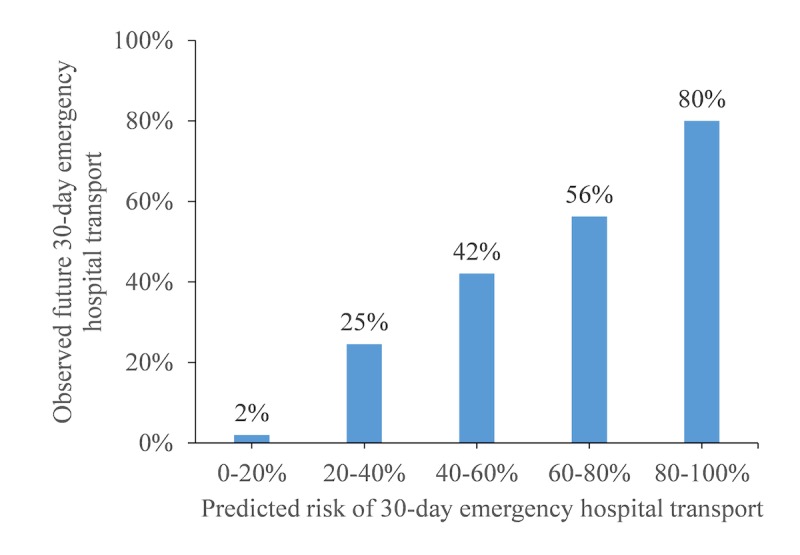
Observed percentage of patients needing 30-day emergency hospital transport versus model-predicted probability in validation cohort 1.

The predictive model produced a probability (from 0% to 100%) for each patient to assess the risk of 30-day emergency hospital transport. The actually observed percentage of patients with emergency hospital transport increased as the predicted probabilities increased (Figure 3). At predicted probabilities between 80% and 100%, 80% (4/5) of patients had 30-day emergency hospital transport in validation cohort 1.

### Predictor Variables

Table 5 provides the number of predictors and total gain, a measure of predictability, for each broad category of predictors. Predictors from the medical alert data formed the most important predictor category for the predictive model, as they accounted for 87.7% of the total gain. A total of 8 predictor variables with zero gain did not contribute to predictability. These included certain uncommon medication allergies and self-reported medical conditions.

**Figure 4:**
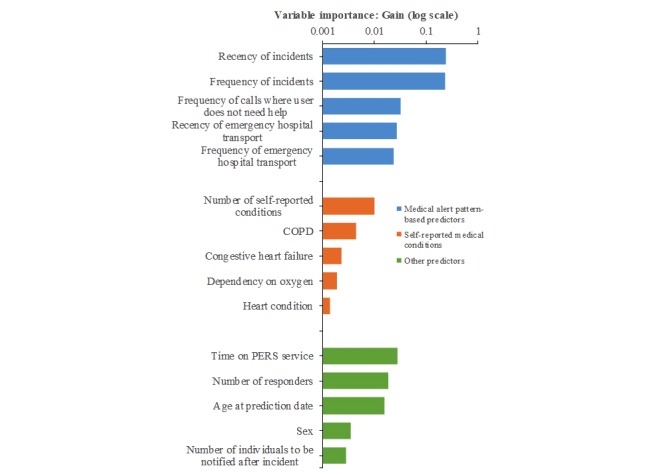
The 5 most important variables in the predictive model for 3 categories of predictors: predictors derived from medical alert data, self-reported medical conditions, and other predictors. Predictor importance as measured by the gain is reported for validation cohort 1. COPD: chronic obstructive pulmonary disease; PERS: personal emergency response system.

The predictive model of 30-day emergency hospital transport included 121 variables with nonzero values for the gain. Figure 4 shows the 5 most important predictors for each category. For self-reported medical conditions, COPD, CHF, and heart conditions are among the 5 most important predictors. Other important predictors include age, sex, and the number of responders.

**Table 5:** Number of predictors and total gain per predictor category.

Predictor category	Number of predictors	Total gain (%)
Medical alert pattern-based predictors	62	87.7
Self-reported medical conditions and medication allergies	44	3.7
Other predictors	15	8.7

### Comparison With Clinical Outcomes

**Figure 5:**
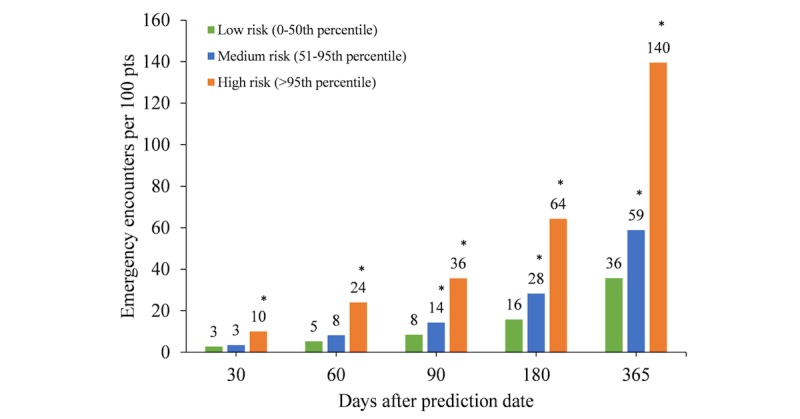
Emergency encounters per 100 patients (pts) in low-, medium-, and high-risk groups in the year following the prediction date. Data shown are for validation cohort 2. *P<.05 compared with low risk, pairwise Wilcoxon rank sum test.

We then used the predictive model of 30-day emergency hospital transport to segment validation cohort 2 into high risk (>95th percentile), medium risk (51st-95th percentile), and low risk (≤50th percentile) after ranking them according to the predicted probabilities of 30-day emergency hospital transport. We normalized the number of emergency encounters (both outpatient visits and inpatient admissions) in the year following the prediction date to 100 patients (Figure 5). Compared with emergency encounters in the low-risk group, patients in the high-risk group had significantly more emergency encounters at 30 (*P*=.001), 60 (*P*<.001), 90 (*P*<.001), 180 (*P*<.001), and 365 (*P*<.001) days. The medium-risk group had significantly more emergency encounters at 90 (*P*=.01), 180 (*P*<.001), and 365 (*P*<.001) days. After 365 days, there were 3.9 times more emergency encounters in the high-risk group than in the low-risk group.

## Discussion

### Principal Findings

Retrospective validation demonstrated the effectiveness of the PERS medical alert data-based predictive model of 30-day emergency hospital transport in identifying patients at risk of hospital transport with good discrimination (AUC=0.779). A goodness-of-fit test and calibration plot indicated that the model-predicted probabilities matched with observed outcomes across ranges of predicted risk. We evaluated the trade-off between finding many true-positive cases (ie, a high sensitivity) and reducing the number of false-positives (ie, a high PPV) by varying the threshold to classify patients as positive. At a risk threshold corresponding to the 99th percentile, 1 out of 4 patients flagged at high risk needed hospital transport in the next 30 days (PPV=25.5%), while at this threshold the sensitivity was rather low at 11.5%. PPV was nearly 12 times higher than the prevalence of 30-day emergency transport in validation cohort 1, which was 2.20%. The trade-off between sensitivity and PPV was also reported by other predictive modeling studies of emergency health care utilization [18,25].

Recent studies have reported on the development and validation of predictive models that can be used to systematically identify individuals at high risk for an unfavorable outcome in a hospital setting [26,27]. Such risk assessment models have great potential to inform treatment decisions and improve the quality of care delivered to patients [26-28]. As innovative connected health technologies and value-based care policy influence the evolution of geriatric models of care to deliver more individualized, multifaceted management strategies, home health programs for community-dwelling seniors may benefit from enhanced risk assessment of patients. For instance, a 32-center randomized controlled trial of the guided care model used health care encounter–based predictive modeling software to identify the 25% of patients with the highest risk of needing complex health care in the coming year. The study provided them with geriatric assessment and specialized support, resulting in significantly reduced health care utilization by home health care patients compared with a group that followed usual care [29]. By using remotely collected PERS data to predict the risk of emergency health care utilization, our predictive model of 30-day emergency hospital transport presents a unique opportunity to efficiently allocate limited health care resources to patients who need them the most and thereby reduce costs associated with excessive use.

This predictive modeling study was, to the best of our knowledge, unique, as it used PERS data to predict emergency hospital transport. Most state laws in the United States require that patients in need of emergency medical care must be taken to the nearest appropriate health care facility capable of treating the patient, which may be an out-of-network facility. Furthermore, in case of ED crowding [30], ambulance diversion to other potentially out-of-network facilities may be requested. In both cases, the resulting ED visit may not be captured in the EHR of the in-network organization. Therefore, predictive models of emergency health care utilization developed on EHR data of a particular health care provider may be missing outcome data. We minimized this limitation in our study, since response agents from the PERS service recorded information about any ambulance transport after an incident that required help from an EMS in the electronic case record of the user.

Linkage of PERS and EHR data in a cohort of 1815 PERS users who were patients of an accountable care organization in the greater Boston area group enabled comparison of PERS–based predicted risk of 30-day emergency hospital transport with emergency encounters derived from the clinical EHR. The patients in the high-risk group—that is, those above the 95th percentile range of risk for emergency transport—also had 3.9 times more emergency encounters per 100 patients in the year following the prediction date than did patients classified as having low risk. Here we specifically analyzed rates of health care encounters, as these are the events that could ultimately be avoided with the appropriate interventions. These results suggest that prediction of emergency health care utilization based on PERS data may be a good alternative to EHR-based prediction models, which could be especially helpful for continuous monitoring of patients after discharge and where patients have missing or limited EHR records.

Our previous study on health care utilization by PERS users indicated that 21% of emergency admissions were considered potentially avoidable [10]. Therefore, we believe that prediction of risk for emergency transport, in combination with appropriate interventions, could potentially reduce emergency hospitalizations. Clinical and financial implications of predictive models largely depend on how well case managers and health professionals can integrate risk prediction of patients into clinical workflows. It is vital to have a detailed guideline that clarifies how the algorithm will inform care [15]. An example of such an approach is a predictive model to detect impending deterioration of patients outside the intensive care unit [31], which was implemented in routine clinical care in 2 community hospitals [32]. In an ongoing randomized clinical trial, we are developing workflows that integrate daily PERS–based risk of 30-day emergency hospital transport with care pathways [33]. In this randomized clinical trial, we are using the predictive model described herein to predict patients’ risk for 30-day emergency transport, followed by a nurse assessment and tailored interventions for high-risk patients. The number of patients who will ultimately benefit from a combination of prediction and intervention will depend on various factors, including the population size and the prevalence of emergency health care utilization, the performance of the predictive model and the risk threshold above which patients are considered to be high risk, and the efficacy of the interventions provided to high-risk patients.

### Limitations

This study had a few limitations. The PERS population is mostly old and primarily female, and the service is predominantly privately paid for by patients and not covered by their health insurance. This may limit the generalizability of the study to older women who can afford the service. Interpretation of the predictive model may have been influenced by confounding of unobserved variables, including when and where users wore the PERS device [34]. While the second objective of the study was to validate the PERS–based prediction model in a specific health system, we believe it is more broadly applicable, since the prediction model was developed on PERS–data only and did not rely on specific inputs from the EHR.

More PERS subscribers in validation cohort 2 self-reported on medical conditions than in the development and validation cohorts. It is likely that this cohort was a biased patient group with higher risk and more medical conditions, as they were receiving care from a health care provider. Given that the AUC for 30-day transport was not significantly lower in validation cohort 2, we do not expect that this affected the predictions. Furthermore, patients with emergency transport in validation cohort 2 may not have ended up in the ED of one of the hospitals of the Partners HealthCare network, but in a different hospital system, such that emergency health care utilization was not recorded in the EHR data that we used. Additionally, patients may have initiated emergency hospital transport outside of the PERS service. Both above-mentioned limitations may have affected the correlation between PERS–based risk for emergency hospital transport and emergency encounters derived from the EHR.

### Conclusions

This study showed that remotely collected patient data using a PERS service can be used to predict 30-day hospital transport. Furthermore, linking these data to clinical observations from the EHR showed that predicted high-risk patients had nearly four times higher rates of emergency encounters in the year following the prediction date compared with low-risk patients. Health care providers could benefit from our validated predictive model by estimating the risk of 30-day emergency hospital transport for individual patients and target timely preventive interventions to high-risk patients. We are testing this hypothesis in a randomized clinical trial where risk predictions are combined with a stepped intervention pathway. This approach could lead to overall improved patient experience, higher quality of care, and more efficient resource utilization. Future studies should explore the impact of combined EHR and PERS data on predictive accuracy.

## Abbreviations

AUC: area under the receiver operating characteristic curve
CHF: congestive heart failure
COPD: chronic obstructive pulmonary disease
ED: emergency department
EHR: electronic health record
EMS: emergency medical service
NPV: negative predictive value
PERS: personal emergency response system
PHH: Partners HealthCare at Home
PPV: positive predictive value
